# Iris-Fixated Intraocular Lenses for Ametropia and Aphakia

**Published:** 2014

**Authors:** Pedro S Simões, Tiago B Ferreira

**Affiliations:** 1 Egas Moniz Hospital, Lisbon, Portugal; 2 Hospital da Luz, Lisbon, Portugal

**Keywords:** Aphakia, Iris-fixated Intraocular Lenses, Phakic Intraocular Lenses, Refractive Errors

## Abstract

Implantation of intraocular lens with Iris-fixation is a safe, efficient and predictable surgical procedure, which empowers the refractive surgeon with singular capabilities. Among their advantages are the reversibility, preservation of accommodation and a broad spectrum of ametropic correction. This lens also appears to be a valid option, with a favorable complication rate, for the treatment of aphakic eyes without capsular support. This article is a review of iris-fixated intraocular lenses and considers their principal indications, complications, and outcomes.

## OVERVIEW

Three types of Phakic intraocular lenses (pIOLs) are commonly used: (I) angle-supported anterior chamber lenses; (II) posterior chamber lenses; and (III) iris-fixated lenses. This review will focus on the latter. Jan Worst developed, in 1978, an iris claw intraocular lens (IOL) for the correction of aphakia following cataract surgery (1). Later, the concept was modified for use in a Phakic eye and the ﬁrst iris-ﬁxated pIOL was implanted by Fechner and Worst in 1986 (2). Iris-fixated pIOLs have two opposed claw haptics that fixate the lens on the iris by enclavation of midperipheral iris stroma, where the iris is less vascularized and less reactive (3). In 1991, the original biconcave Worst myopia claw lens was changed into the convex–concave shape. This design decreased the potential for complications, improved the optical performance and simplified the implantation technique. In 1992, the first hyperopia lens model was implanted (4). In 1998, the name of the lens changed to Artisan lens (Ophtec B.V., Groningen, the Netherlands), Verisyse in the US (5). The Artisan iris-fixated lens is a one-piece all polymethyl methacrylate (PMMA) IOL. The total length of the lens is 8.5 mm with an optic of 5 or 6 mm in diameter. Since 1999, the Artisan lens is also available for astigmatism correction. In 2003, the foldable iris-ﬁxated Artiﬂex pIOL (Ophtec B.V.), Veriflex in the US, became available (5). This model was based on the Artisan platform, with a flexible, convex-concave, 6 mm silicone optic, PMMA haptics and overall length of 8.5 mm (6). The added value of the ﬂexible Artiﬂex over the Artisan is the small incision through which it can be inserted (3.2 mm), decreasing surgically induced astigmatism and accelerating visual recovery (5, 6). Since 2009, the Artiflex lens is also available in Europe for astigmatism correction. Table 1 summarizes iris-fixated pIOLs specifications.

**Table 1:** Available Iris-fixated pIOLs specifications. Adapted from Alio, J.L. and Perez-Santonja (7).

**Model**	**Material**	**Power (D)**	**Optic diameter (mm)**
**Artisan/ Verisyse**	PMMA, one-piece	Myopia -1 to -15.5	6
		Myopia -1 to -23.5	5
		Hyperopia +1 to +12	5
		Toric +6 to -23, torus +1 to +7	5
		Aphakic +2 to +30	5.4
**Artiflex / Veriflex**	Polysiloxane optic; PMMA haptics	Myopia -2 to -14.5	6
		Toric -1 to -13.5, torus -1 to -5	6

## REFRACTIVE ERROR

Ametropias are the most common eye problem in the US. Among individuals over age 40, 30.5 million have significant myopia and 12 million visually suffer from high hyperopia. Other common refractive errors include astigmatism and presbyopia (8, 9). Refractive surgery is nowadays a valid alternative to treating a broad range of refractive errors and, consequently, the number of people seeking this care is likely to increase (8, 10).

Various surgical modalities and techniques exist to treat a broad range of refractive errors. Laser corneal refractive surgery is effective, safe and has been the favored option for refractive surgery over the last decade (11). However, restrictions imposed by corneal thickness, curvature or pupil size make it inadequate to treat some ametropic eyes, particularly those with high refractive errors (12, 13).

Refractive lens exchange with implantation of an appropriate posterior chamber IOL can correct the ametropia. Though, lensectomy results in a complete loss of accommodation for pre-presbyopic patients and carries some retinal risks, especially, in patients with high myopia (14, 15). Iris-fixated pIOLs increase the capabilities of refractive surgery as they have the potential to correct any ametropia, including hyperopia and astigmatism. Their power does not depend on tissue healing, and their effect is reversible. Furthermore, the crystalline lens remains intact, and accommodation is preserved (15, 16). Some authors have proposed an alternative methodology, combining corneal and intraocular procedures to improve results in high ametropia (17). This approach called bioptics, consists in IOL implantation followed by laser corneal refractive surgery and offers a different surgical option to correct important refractive errors (18). Iris-fixated pIOLs might take part in bioptics as well. Phakic IOLs have also been proposed as a surgical option to correct astigmatism and spherical ametropia in contact lens intolerant keratoconus patients (19). As a combined treatment, iris-fixated pIOLs have a significant role in the management of residual spherocylindrical refractive errors, after other keratoconus treatment modalities have been used, namely, corneal collagen cross-linking (20) or intracorneal ring segments (ICRS) (21).

## ARTISAN / VERISYSE

Follow-up of the Artisan pIOL report that correction of moderate to high myopia is a stable, predictable and safe method, when inclusion criteria for surgery are applied (22). Different studies stated that stabilization of the postoperative refraction occurs in the early years after implantation, the great majority of the eyes achieve a refraction within 1.0 D of the intended correction and more than 60% of the patients gained two or more Snellen lines of corrected distance visual acuity (CDVA). Complications are infrequent and rarely cause loss of corrected visual acuity (14,23-25).

High hyperopia is a challenge in refractive surgery. Although the predictability of the refractive results appears to be lower than those in the correction of myopia and astigmatism (as with other surgical procedures for hyperopia), Artisan or Artisan toric pIOLs can correct moderate to high hyperopia, combined or not with astigmatism, with good refractive results. Still, its use is restricted by the anterior chamber depth (ACD), of paramount importance for moderate and high hyperopia, which is typically accompanied by a shallow anterior chamber (26,27). The FDA clinical trial set the inferior ACD limit of 3.2 mm, although 3.0 mm is the value recommended by the manufacturer (14). Table 2 summarizes the additional inclusion criteria in the study.

**Table 2:** Inclusion Criteria – US FDA Artisan / Verisyse Clinical Trial. Adapted From Stulting, R. D., et al. (14)

**Age**	**21 – 50**
**Axial myopia**	4.5 D – 22.0 D (Stable manifest refraction)
**Astigmatism**	≤ 2.0 D (Spectacle plane)
**ACD**	≥ 3.2 mm
**Pupil size**	≥ 4.5 mm (Ambient light)
**Endothelial cell count**	≥ 2000 cells/mm^2^

Phakic intraocular lenses are implanted in a relatively young population, and the possibility of progressive endothelial cell loss over a lifetime is of concern (14). Nevertheless, there is strong evidence in the literature, that the current Artisan Phakic IOL design is long-term stable, and the implanted lens does not continue to stress the endothelium (14, 23). Complications are uncommon and appear to be related to the learning curve, occurring when surgeons are relatively inexperienced (14). Glare levels, accessed using a validated questionnaire, are low. Moreover, overall satisfaction after Artisan pIOL implantation for myopia is excellent (28).

**Figure 1:**
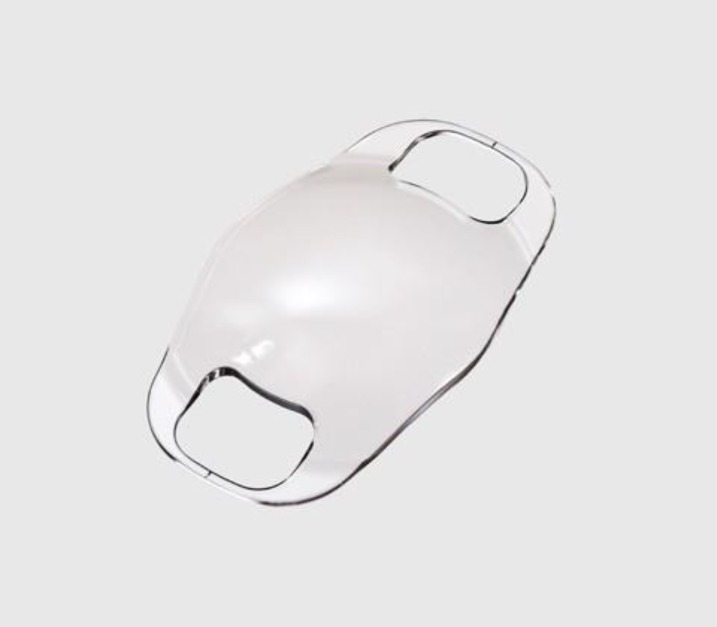
Artisan / Verisyse Myopia (3).

Refractive surgery is controversial in the pediatric population. However, it can be useful for cases of amblyopia with poor compliance or when conventional treatments are unsuccessful, such as high anisometropia and reduced compliance owed to social circumstances or neurobehavioral disorders (29, 30). Though more robust studies are needed, pIOL implantation appears to be an effective option in severe cases of anisometropia associated to amblyopia in children, in whom conventional treatment methods are not adequate and laser refractive surgery is not suitable (30) (Figure 1).

## ARTIFLEX / VERIFLEX

Modern refractive surgery’s standard of minimally invasive procedures through minor, sutureless wounds, lead into the development of foldable lenses.

**Figure 2:**
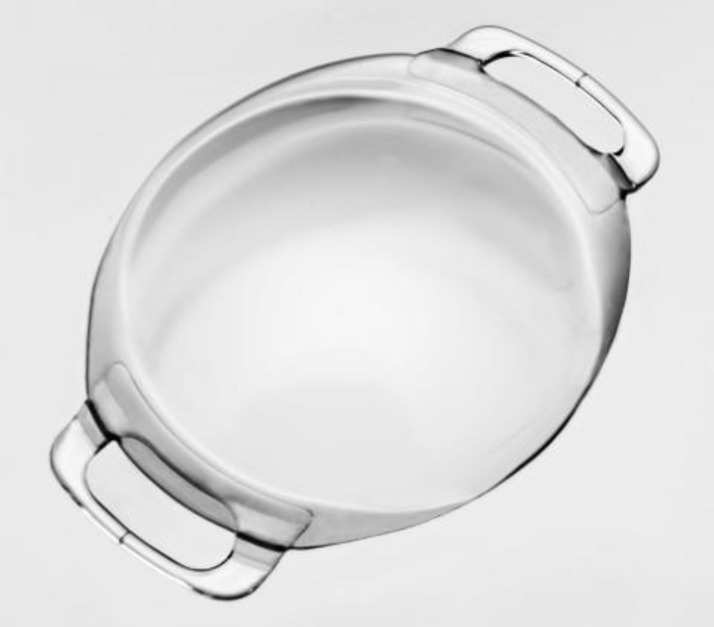
Artiflex / Veriflex Myopia (3)

Small incisions grant faster visual recovery with less induced astigmatism (6). As stated before, the Artiflex is a foldable version of the Artisan pIOL, with a similar design (Figure 2). Outcomes are mostly comparable with those of previous Artisan studies (31). Though a higher occurrence of pigment precipitates when compared to the Artisan lens has been reported, in most cases they appear to be transient and without clinical significance (6,31). Artiflex pIOL appears to be a useful improvement of the Artisan product family with excellent predictability, efficacy, and safety, when speciﬁcations of the manufacturer are taken into account regarding patient selection and postoperative care (6,32). As with the Artisan lens, the author´s experience endorse the importance of the morphometric study of the anterior chamber (AC) during the follow-up period (33).

## APHAKIA

Following cataract surgery, best result is achieved with the implantation of an IOL in the capsular bag. However, several conditions can lead to aphakia in eyes with insufficient or absent capsule support. Among others, these include crystalline lens subluxation, IOL dislocation, capsular loss during cataract extraction for congenital or juvenile cataract, complicated phacoemulsification for senile cataract and trauma (34,35). The resulting aphakia can be corrected with aphakic spectacles, contact lenses, and implantation of aphakic IOLs (35,36.).

Results with spectacles are often poor because of prismatic effects, image magnification, aberrations of images, a limited vision field, and appearance concerns (37). Contact lenses provide better visual results than spectacles; yet, they are associated with a higher risk for microbial keratitis and corneal erosion (38).

Options to surgically correct aphakia include implantation of a transsclerally sutured posterior chamber (PC) IOL (39), angle-supported AC IOL (40), or an iris-fixated IOL (41). Angle-supported AC IOLs are rarely used because of the high incidence of secondary glaucoma, pupil distortion, endothelial cell loss, and IOL instability (42).

Transsclerally sutured PC IOLs preserve the anterior chamber anatomy and cause less endothelial damage than angle-supported AC IOLs. However, they present a high incidence of intraoperative and postoperative complications. In addition, the transscleral ﬁxation of a PC IOL is a technically challenging procedure, requiring a long surgical time and having a steep learning curve (42,43).

**Figure 3:**
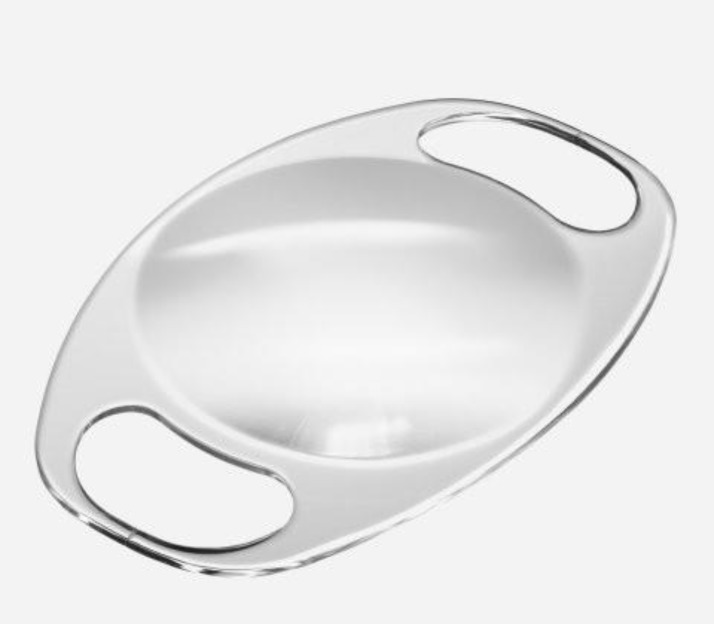
The Artisan aphakia IOL presents a biconvex design with ultraviolet light filtration. The surgeon implants the IOL by placing it on the iris, centering on the pupil and clutching a fold of midperipheral iris stroma, without interfering with iris vascularization or the trabecular meshwork. They are marginally raised above the iris plane, except at the ﬁxation points, preventing them from meddling with the normal physiology of the iris or angle structures. These IOLs are used to correct aphakia (a similar model is used to correct refractive errors) (14,24)

Iris-fixated IOLs (view Table 1 for specifications) are easy to place, yield favorable visual outcomes and have a lower incidence of intraoperative and postoperative complications when compared with the previous IOL types (14,43,44). Distinct studies report safe implantation of these IOLs even in the pediatric population (45) (Figure 3).

Primary concern with Artisan aphakic iris-fixated IOL implantation is corneal endothelial cell loss (33). This is of great significance in children, considering their long life expectancy (44). Although more prospective studies of corneal endothelium outcomes, particularly in the young population, are needed, the corneal endothelial cell loss seems to be the result of mechanical injury during surgery (34). Furthermore, mean corneal endothelial cell density (ECD) was comparable to the mean normal ECD in the same age group reported in the literature (45). Another concern is the potential damage to the iris (34). However, the Artisan IOL design provides enough clearance between the IOL and the iris, preventing the IOL from interfering with the normal physiology of the iris, as long as the IOL is properly implanted (46). Other infrequent intraoperative and postoperative complications reported with these IOLs include IOL dislocation, pupillary block glaucoma (ensuring a patent iridotomy is present is of paramount importance in any iris-fixated pIOL), retinal detachment, pupil ovalization, hyphema, and cystoid macular edema (23,47).

Implantation of an Artisan aphakic iris-fixated IOL is supported by different studies, which report promising results and excellent safety, efficacy, predictability and stability for the correction of aphakia in eyes with insufficient capsular support (43,47,48).

## CONCLUSION

Modern iris-fixated intraocular lenses are the result of continuing technological evolution over the last 30 years. Their implantation appears a safe, predictable and stable surgical procedure, which offers a broad range of refractive errors correction and reversibility. Aphakia can be corrected with a favorable complication rate. Although the concern has been raised about corneal endothelial loss and a close follow-up is advisable, no reliable evidence puts in danger iris-fixated IOL implantation, when speciﬁcations of the manufacturer are taken into account. Sequential use of pIOLs after additional refractive procedures may result in a better visual outcome in selected cases.
